# Effects of various acute hypoxic conditions on metabolic parameters and cardiac function during exercise and recovery

**DOI:** 10.1186/s40064-016-2952-4

**Published:** 2016-08-08

**Authors:** Hwang-woon Moon, Sub Sunoo, Hun-young Park, Dong-jun Lee, Sang-seok Nam

**Affiliations:** 1 Department of Sports and Outdoors, Eulji University, Yangji-dong, Sujeong-gu, Seongnam-si, Gyeonggi-do 461-713 Republic of Korea; 2 Department of Sports Medicine, Kyunghee University, 1732, Deogyeong-daero, Giheung-gu, Yongin-si, Gyeonggi-do 17104 Republic of Korea; 3 Performance Activity and Performance Institute, Konkuk University, Hwayang-dong, Gwangjin-gu, Seoul, 143-701 Republic of Korea; 4 Department of Physical Education, MyongJi University, Yongin Campus, Nam-dong, Cheoin-gu, Yongin-si, Gyeonggi-do 449-728 Republic of Korea

**Keywords:** Normobaric hypoxic condition, Oxygen partial pressure, Metabolic parameters, Cardiac function, Aerobic exercise capacity

## Abstract

**Purpose:**

Evaluation of metabolic parameters and cardiac function is important to determine the decrease in aerobic exercise capacity under hypoxic conditions. However, the variations in metabolic parameters and cardiac function and the reasons for the decrease in aerobic exercise capacity under hypoxic conditions have not been clearly explained. The purpose of this study was to compare the responses between sea level and various acute normobaric hypoxic conditions on metabolic parameters and cardiac function during exercise and recovery in order to evaluate aerobic exercise capacity.

**Methods:**

Ten healthy male participants (21.3 ± 3.06 y) performed submaximal bicycle exercise (116.7 ± 20.1 W and 60 rpm) at sea level (20.9 % O_2_) and under various normobaric hypoxic conditions (16.5 % O_2_, 14.5 % O_2_, 12.8 % O_2_, and 11.2 % O_2_) in a random order. Metabolic parameters (arterial oxygen saturation; S_P_O_2_, oxygen consumption; VO_2_, blood lactate level) and cardiac function (heart rate; HR, stroke volume; SV, end-systolic volume; ESV, end-diastolic volume; EDV, ejection fraction; EF, cardiac output; CO) were measured at rest, during exercise (30 min), and recovery (30 min). We compared the responses on metabolic parameters and cardiac function between the different oxygen partial pressure conditions during exercise and recovery.

**Results:**

The various acute normobaric hypoxic conditions did not affect VO_2_ and SV during exercise and recovery. S_P_O_2_ decreased (*p* < .05) and blood lactate level increased (*p* < .05) as the oxygen partial pressure decreased. HR, EF, CO increased (*p* < .05) and EDV, ESV decreased (*p* < .05) at oxygen partial pressures of 14.5 % O_2_ and below compared with 20.9 and 16.5 % O_2_ during exercise and recovery.

**Conclusion:**

A decrease in the oxygen partial pressure to 14.5 % O_2_ and below might be associated with significant changes in metabolic parameters and cardiac function during exercise and recovery. These changes are an acute compensation response to reduced aerobic exercise capacity by decreased oxygen delivering and utilizing capacities under hypoxic conditions.

## Background

In 1968, the Olympic Games were held at high altitude in Mexico City, and since then, the effects of hypoxic conditions at high altitude on exercise performance have received considerable attention (Morton and Cable 2005; Hinckson et al. 2006).

Under hypoxic conditions, oxygen partial pressure decreases owing to low atmospheric pressure, and this results in the reduction of the alveolar oxygen partial pressure, arterial blood oxygen saturation (S_p_O_2_), and arteriovenous oxygen difference, diminishing the capacity for oxygen delivery and utilization (Bhaumik et al. 2008; Brutsaert 2008). Compared with sea level (normoxia), submaximal exercise at the same intensity in hypoxic condition causes increase in ventilation, heart rate (HR), cardiac output (CO), blood lactate levels, and oxygen consumption (VO_2_) due to decrease in oxygen delivering and utilizing capacity of the blood (Mazzeo et al. 1991; Welsman et al. 2005). In addition, maximal oxygen consumption (VO_2_max) and maximal power output are reduced in hypoxic conditions compared with sea level (Lawler et al. 1988; Robergs et al. 1998). Therefore, these results may shed light on metabolic parameters and cardiac function to response to various hypoxic conditions during exercise. Previous studies investigated the decrease in aerobic exercise capacity (e.g. HR, SpO_2_, minute ventilation, red blood cell; RBC count, hemoglobin; Hb level, hematocrit; Hct, and VO_2_) under hypoxic conditions during submaximal exercise and VO_2_max (Lawler et al. 1988; Calbet et al. 2003; Friedmann et al. 2007; Bhaumik et al. 2008). However, very few studies conducted the effect of acute hypoxia on cardiac function. Most previous studies reported that acute exposure to hypoxic conditions activates the sympathetic nervous system and increases HR (Hainsworth and Drinkhill 2007; Yan et al. 2007; Fukuda et al. 2010). However, results have been inconsistent regarding stroke volume (SV), end-systolic volume (ESV), end-diastolic volume (EDV), ejection fraction (EF), systemic vascular resistance (SVR), and CO (Thomson et al. 2006; Hsu et al. 2006; Hainsworth and Drinkhill 2007; Yan et al. 2007; Fukuda et al. 2010). These inconsistencies might have resulted from differences in the study design, extent of hypoxia, exercise type, intensity, and duration (Thomson et al. 2006; Lawler et al. 1988; Sunoo et al. 1997; Morton and Cable 2005; Hinckson et al. 2006; Friedmann et al. 2007; Adamos et al. 2008; Elstad et al. 2009). Therefore, variations in metabolic parameters and cardiac function, and decrease in aerobic exercise capacity under hypoxic conditions have not been explained clearly.

The present study was conducted to clarify the effects of acute normobaric hypoxic conditions on metabolic parameters and cardiac function during exercise and recovery in order to evaluate change on aerobic exercise capacity according to various hypoxic conditions.

## Methods

### Participants

The present study included 10 healthy college males who did not participate in any planned exercise program and did not consume any dietary supplements in the previous 6 months. The participants were non-smokers, and did not have any history of musculoskeletal, cardiovascular, or pulmonary disease. They received information about the purpose and process of this study, and provided informed consent prior to the start of the study. Characteristics of the participants are presented in Table 1. This study was approved by the Institutional Review Board of Kyung Hee University (KHSIRB 2015-020) in Korea and was conducted according to the declaration of Helsinki.

**Table 1:** Characteristic of participants

Variables	Descriptive statistics	Variables	Descriptive statistics
Number (n)	10	Fat free mass (kg)	35.5 ± 4.41
Age (years)	21.3 ± 3.06	Fat mass (kg)	11.3 ± 5.04
Height (cm)	176.9 ± 4.38	Body mass index (kg/m^2^)	22.5 ± 2.40
Weight (kg)	70.8 ± 9.68	Body fat (%)	15.5 ± 4.98

### Experimental design

All participants performed a physical work capacity (PWC) test at 70 % HRmax to determine their bicycle exercise intensity at sea-level (20.9 % O_2_) and HRmax was determined using the predicted HRmax formula (Male = 206 − 0.69 × age, Miyashita et al. 1985). The Aerobike 75XL II (Combi Corporation, Tokyo, Japan) was used to measure PWC 70 % HRmax. All participants performed submaximal exercise at 116.7 ± 20.1 W and maintained 60 rpm during 30 min exercise at sea level (20.9 % O_2_) and various normobaric hypoxic conditions (16.5 % O_2_, 14.5 % O_2_, 12.8 % O_2_, and 11.2 % O_2_) in a random order. Right after exercise, participants were held still on a bicycle for 30 min during recovery. The variables were measured at rest, during exercise (30 min), and recovery (30 min post exercise) at sea level and various normobaric hypoxic conditions.

The exercise performed in a 6.5 × 7.5 × 3 m environmental chamber (Submersible Systems, Huntington Beach, CA) for all the conditions. The various normobaric hypoxic conditions simulated by introducing nitrogen into the environmental chamber, using a nitrogen generator (Separation & Filter Energy Technology Cooperation, Siheung, Korea) with the capacity to simulate normobaric hypoxic conditions for altitudes of up to 9.7 % O_2_ (6000 m). The temperature and humidity within the environmental chamber were maintained at 20 ± 2 °C and 60 ± 2 % for all the conditions, respectively. Each participant performed the bicycle exercise at sea level and various normobaric hypoxic conditions, and a minimum interval of 7 days was ensured between conditions.

### Cardiometabolic measurements

The index finger of each participants were placed on the S_p_O_2_ sensor of a radical-7 pulse oximeter (Masimo Corporation, Irvine, CA) to measure S_p_O_2_. The Vmax-229, breath-by-breath automatic metabolism analyzer (SensorMedics, Yorba Linda, CA) and a breathing valve in the form of a facemask used to measure VO_2_ at rest, during exercise, and recovery under all the hypoxic conditions. For measuring the blood lactate level, we collected 80 μL of blood in a capillary tube using the fingertip method, and the sample analyzed using the YSI-1500 lactate analyzer (YSI Inc., Yellow Springs, OH). Cardiac function parameters, including the HR, SV, EDV, ESV, EF, and CO were assessed noninvasively by using a thoracic bioelectrical impedance device (PhysioFlow PF-05 Lab1, Manatec Biomedical, Paris, France), which has been previously shown to provide reliable results in healthy men and patients with chronic pulmonary disease (Charloux et al. 2000; Fukuda et al. 2010). The electrodes positioned on the forehead, neck, xiphoid process, and lower ribs on the left side, avoiding the abdominal muscles, as these positions were suggested to be appropriate for human participants (Charloux et al. 2000; Fukuda et al. 2010).

### Statistical analysis

All data are presented as means ± standard deviations. Two-way repeated analysis of variance (ANOVA) was used to figure out the interaction effects between condition and time at rest, during exercise, and recovery. Repeated one-way ANOVA was used to evaluate differences in dependent variables between all the conditions at each time point and post hoc test was used LSD (least significant difference). All statistical analyses were performed using SPSS version 22.0 (IBM Corp., Armonk, NY) for Windows. The level of significance was set at *p* < .05.

## Results

**Fig. 1:**
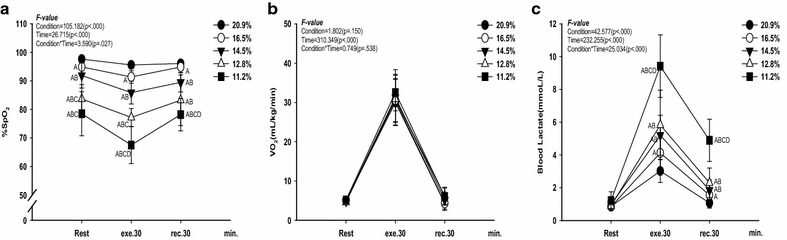
Changes in metabolic parameters at rest, exercise time, and recovery time for all the conditions. **a** change in S_P_O_2_ (%). **b** change in VO_2_ (mL/kg/min). **c** change in blood lactate level (mmol/L). The bars indicate the mean ± S.D. ^A^ *p* < .05 vs. 20.9 % O_2_, ^B^ *p* < .05 vs. 16.5 % O_2_, ^C^ *p* < .05 vs. 14.5 % O_2_, ^D^ *p* < .05 vs. 12.8 % O_2_

**Fig. 2:**
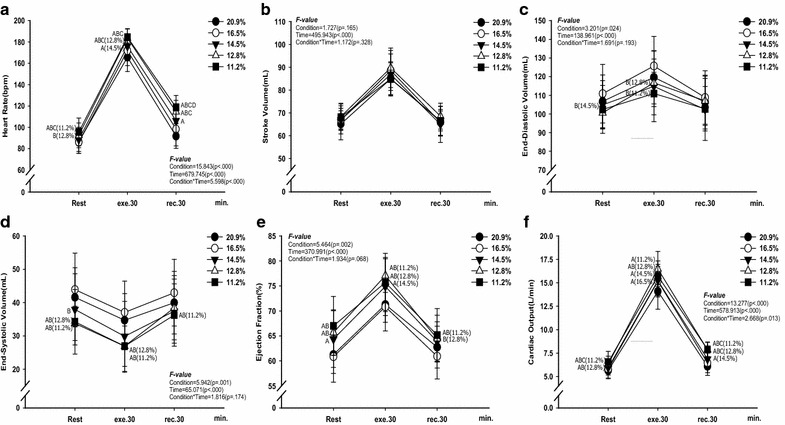
Changes in cardiac function at rest, exercise time, and recovery time for all the conditions. **a** change in HR (bpm). **b** change in SV (mL). **c** change in EDV (mL). **d** change in ESV (mL). **e** change in EF (%). **f** change in CO (L/min). The bars indicate the mean ± S.D. ^A^ *p* < .05 vs. 20.9 % O_2_, ^B^ *p* < .05 vs. 16.5 % O_2_, ^C^ *p* < .05 vs. 14.5 % O_2_, ^D^ *p* < .05 vs. 12.8 % O_2_

There were some changes in metabolic parameters and cardiac function at rest, during exercise, and recovery as it is shown in Figs. 1 and 2.

### Metabolic parameters

As shown in Fig. 1, there were interaction effects between condition and time on S_P_O_2_ (F = 3.590, *p* = .027, panel a) and blood lactate level (F = 25.034, *p* < .000, panel c). S_P_O_2_ decreased (*p* < .05) as the oxygen partial pressure decreased at rest, during exercise, and recovery. Blood lactate level increased at 16.5 % O_2_, 14.5 % O_2_, 12.8 % O_2_, and 11.2 % O_2_ (*p* < .05) compared with 20.9 % O_2_ during exercise and recovery, and it was increased at 11.2 % O_2_ (*p* < .05) compared with all other oxygen levels. However, there was no difference in VO_2_ between all the conditions.

### Cardiac function

As shown in Fig. 2, there were interaction effects between condition and time on HR (F = 5.598, *p* < .000, panel a) and CO (F = 2.668, *p* = .013, panel f). HR and CO increased in the oxygen partial pressure to 14.5 % O_2_ and below (*p* < .05) compared with 20.9 % O_2_ during exercise and recovery (panels a and f) but SV showed no changes between all the conditions (panel b). EDV and ESV decreased at 12.8 % O_2_, and 11.2 % O_2_ (*p* < .05) compared with 20.9 % O_2_ during exercise (panels c and d). EF increased in the oxygen partial pressure to 14.5 % O_2_ and below (*p* < .05) compared with 20.9 % O_2_ during exercise and recovery (panel e).

## Discussion

The present study was conducted to investigate the responses between various acute normobaric hypoxic conditions on metabolic parameters and cardiac function during exercise and recovery in order to evaluate aerobic exercise capacity. Our findings indicate that S_P_O_2_ decreased (p < .05) and blood lactate level increased (p < .05) as the oxygen partial pressure decreased but that VO_2_ and SV were not affected by the various acute normobaric hypoxic conditions. In addition, HR, EF, and CO increased, but EDV, ESV decreased at oxygen partial pressures of 14.5 % O_2_ and below compared with 20.9 and 16.5 % O_2_ during exercise and recovery.

### Metabolic parameters

Acute exposure to hypoxic conditions increases oxygen demand by lowering the capacity of the blood to transport oxygen and that of muscle to utilize oxygen, resulting in decrease in VO_2_ at the same relative intensity, as well as a decrease in VO_2_max (Lawler et al. 1988; Wehrlin and Hallén 2006). In our study, S_p_O_2_ decreased as oxygen partial pressure decreased at rest, during exercise and recovery. However, we did not observe any significant differences in VO_2_ between sea-level and various hypoxic conditions during submaximal exercise and recovery, this is likely because the exercise intensity was fixed at constant level across simulated environment conditions resulting in the same amount of energy expenditure. Concordant with previous studies, increases in ventilation, breathing rate, HR, and cardiac output adapted to acute hypoxic environments were possible without an increase in VO_2_ (Hill et al. 2011; Mazzeo 2008). In addition, our findings are consistent with those reported previously by Adamos et al. (2008) and Calbet et al. (2003). These studies found no significant differences in VO_2_ during submaximal exercise between a simulated 5300 m hypoxic condition and sea level. The absence of an effect on VO_2_ may be explained by changes in cardiac function, specifically increases in HR, SV, and CO in response to reduced oxygen delivery and utilization capacities of the blood. Such changes in cardiac function were evident in our study group, and there were significant differences in HR, SV, ESV, and CO between sea level and the various hypoxic conditions.

In addition, blood lactate level increased as oxygen partial pressure decreased corresponding to increased levels in the present study. Under hypoxic conditions, decreased cardiometaboic levels (e.g. oxygen delivery, utilization of capacity of blood, and arterio-venous O_2_ difference) may result in an increase in the anaerobic energy metabolism contrast to decrease in the aerobic energy metabolism. For example, Adamos et al. (2008) reported that an increase in blood lactate level indicated the dependence of ATP resynthesize system on the anaerobic process. Additionally, decrease in oxygen delivery and utilizing capacities were associated with an increase in blood lactate level and led to increase in lactate production according to increase in lactate appearance and decrease in lactate disappearance during exercise (Park et al. 2016).

### Cardiac function

Our findings demonstrate that HR increased during exercise and recovery as the oxygen partial pressure decreased; however, VO_2_ did not change. Under hypoxic conditions, HR increases to compensate for the decrease in oxygen delivery and utilizing capacities, and the activated sympathetic nervous system (Holloway et al. 2011). A previous study showed that the sympathetic nervous system is activated by stimulation of afferent nerve in the metabolic receptor “Group IV” from accumulation of metabolites such as lactate and hydrogen ions (Sausen et al. 2009). Adamos et al. (2008) reported that HR increases by activation in the sympathetic nervous system and increased catecholamine level during exercise and recovery under hypoxic condition. Hainsworth and Drinkhill (2007)reported that activation of the sympathetic nervous system stimulates the release of catecholamine from the adrenal medulla under hypoxic conditions. Mazzeo (2008) stated that the stimulation of β-adrenaline receptors is a major factor responsible for the increase in HR during exercise under hypoxic conditions. Additionally, EDV and ESV are major factors affecting SV. A previous study reported that an increase in EDV and decrease in ESV could result in increases in ventricular contractility and SV (Hsu et al. 2006). In the present study, EDV increased at 12.8 % O_2_, and 11.2 % O_2_ compared with 16.5 % O_2_ during exercise. However, EDV showed no differences between hypoxic conditions and 20.9 % O_2_. ESV decreased at 12.8 % O_2_ and 11.2 % O_2_ compared with 20.9 % O_2_ during exercise. Therefore, compared with 20.9 % O_2_, EF (calculated as SV/EDV) increased in the oxygen partial pressure to 14.5 % O_2_ and below during exercise and increased in the oxygen partial pressure to 12.8 % O_2_ and below during recovery. These results may indicate that the activation of sympathetic nervous system and catecholamine level of the blood are triggered by increased calcium ions in cardiac muscle cells during exercise under hypoxic conditions (Mason 2000). However, in the present study, SV did not change between conditions. Also, this result might be explained by the reduction in left ventricular expansion capacity during exercise under hypoxic conditions (Fukuda et al. 2010). CO increased by HR during exercise and recovery as the oxygen partial pressure decreased. This is an acute compensation response for decrease in oxygen diffusion, delivery, and utilizing capacities in the lungs and muscle tissues (Naeije 2010).

## Conclusion

A decrease in the oxygen partial pressure to 14.5 % O_2_ (3000 m normobaric hypoxic condition) and below might be associated with decreases in S_p_O_2_, EDV, ESV and increases in blood lactate level, HR, EF, and CO during submaximal exercise. These changes are an acute compensation response to reduced aerobic exercise capacity by decreased oxygen delivering and utilizing capacities under hypoxic conditions. However, a decrease in the oxygen partial pressure might not have an influence on SV.

### Limitation

There are several limitations in the present study that should be noted. First, all participants were young healthy individuals, so a complete cross-section of the population was not represented. Second, the sample size was too small for sufficient power for some variables. Moreover, not all possible variables that might affect aerobic exercise capacity were investigated. A follow-up review with an increased sample size, including the same study design with the addition of other related factors is needed to further investigate the impact of high altitudes (hypoxic conditions) on exercise in relation to changes in various dependent variables.
